# Exercise Restriction Does Not Change Outcome in Dogs After Diagnosis of Acute Non‐Compressive Nucleus Pulposus Extrusion, Fibrocartilaginous Embolism, or Hydrated Nucleus Pulposus Extrusion

**DOI:** 10.1111/jvim.70135

**Published:** 2025-06-03

**Authors:** Katherine Phillips, Paul Freeman

**Affiliations:** ^1^ Department of Clinical Veterinary Medicine Queen's Veterinary School Hospital, University of Cambridge Cambridge UK

**Keywords:** fibrocartilaginous infarct, intervertebral disc disease, neurology, spinal cord disease

## Abstract

**Background:**

Acute non‐compressive nucleus pulposus extrusion (ANNPE), fibrocartilaginous embolism (FCE), and hydrated nucleus pulposus extrusion (HNPE) all present with acute to peracute onset of myelopathic signs in dogs, for which treatment usually is medical. Conservative management including a period of strict rest usually is recommended because of concern about relapse if immediate exercise is allowed.

**Hypotheses:**

1. Allowing exercise after a diagnosis of ANNPE, FCE, or HNPE does not decrease the chance of recovery or predispose to relapse in the short‐term follow‐up period (4 weeks) after discharge.
2. There is no difference in clinical outcome between rested and exercised groups.

**Animals:**

Forty cases of ANNPE, FCE, or HNPE with follow‐up including at minimum a 4‐week evaluation after discharge.

**Methods:**

Retrospective study. The exercise group (*n* = 22) included all dogs the owners of which were not explicitly instructed to rest their dogs, whereas the rest group (*n* = 18) included all dogs the owners of which were specifically advised to rest their dogs or to restrict their exercise.

**Results:**

No dogs relapsed or deteriorated between discharge and 4‐week re‐evaluation. No significant difference was found between the rest and exercise groups.

**Conclusions:**

Allowing exercise after confirming a diagnosis of ANNPE, FCE, or HNPE did not predispose to relapse of clinical signs in the 4 weeks after discharge in our cohort, but the rest group had a higher proportion of HNPE cases, which means interpretation of our findings in this diagnosis must be made with caution.

**Abbreviations**
AF: annulus fibrosus
ANNPE: acute non‐compressive nucleus pulposus extrusion
FCE: fibrocartilaginous embolism
HNPE: hydrated nucleus pulposus extrusion
IVD: intervertebral disc
MRI: magnetic resonance imaging
NP: nucleus pulposus
QVSH: Queens Veterinary School Hospital

## 1 Introduction

Acute non‐compressive nucleus pulposus extrusion (ANNPE) and fibrocartilaginous embolism (FCE) present as a peracute onset of usually non‐painful myelopathic signs in dogs and for which treatment is generally considered to be non‐surgical [1, 2]. Many investigations do not attempt to separate these conditions because diagnosis is difficult even with magnetic resonance imaging (MRI) [3], and they are similar in presentation, treatment, and prognosis. Hydrated nucleus pulposus extrusion (HNPE) cases also present in a very similar way [3].

Acute non‐compressive nucleus pulposus extrusion often is preceded by vigorous exercise or trauma leading to the herniation of non‐degenerate nucleus pulposus (NP) through the ruptured annulus fibrosus (AF) causing a contusive injury to the spinal cord [4, 5, 6, 7]. Characteristic presenting signs represent a peracute, often lateralizing and severe myelopathy, variable in neuroanatomical localization [4, 5, 7, 8]. This presentation is very similar to that of FCE [2]. Generally, dogs with ANNPE and FCE have non‐progressive diseases or improve and become pain‐free after 24 h [2, 8].

Cases of HNPE usually present acutely and are caused by herniation of non‐ or minimally degenerate NP through a ruptured AF [4]. However, in these cases a degree of spinal cord compression often is present [9]. The lesions more commonly affect the cervical spinal cord and are less often lateralized. Management may be conservative or surgical, but no difference in outcome has been reported [2, 4, 9, 10].

Diagnosis of these conditions requires MRI with similarities especially between ANNPE and FCE [6, 9, 10, 11]. Given that the majority of these patients do not undergo surgery or euthanasia, a combination of clinical history, physical and neurological examination with or without MRI is used to make a presumptive diagnosis [2, 4, 6, 7, 8, 11].

Most studies of these conditions report that the affected animals were strictly rested for 4–6 weeks after initial presentation and diagnosis [2, 4, 5, 6, 7, 8, 9, 10, 11, 12]. A previous study described a neurorehabilitation regimen implemented on Day 2 after a confirmed ANNPE diagnosis [13]. There has been little discussion on the value of cage rest in these cases, despite the burdens on animals and their caregivers of such a regimen [14, 15]. Our aim was to evaluate if an increased chance of relapse was associated with owners not being explicitly told to enforce exercise restriction of their affected dogs.

## 2 Methods

A search of the imaging database of Queen Veterinary School Hospital (QVSH), University of Cambridge, between 2012 and 2023 was made based on affected dogs using the following key words: acute non‐compressive nucleus pulposus extrusion, high velocity low volume, fibrocartilaginous embolism, hydrated nucleus pulposus extrusion, and spinal cord hyperintensity. A file search of the hospital records also was performed using the search terms: acute non‐compressive nucleus pulposus extrusion, ANNPE, fibrocartilaginous embolism, FCE, hydrated nucleus pulposus, and HNPE. Inclusion criteria were dogs presented with peracute (< 6 h) or acute (< 24 h) onset (consistent with ANNPE, FCE, HNPE) static or improving myelopathy [2]; MRI to confirm suspected diagnosis of ANNPE, FCE, or HNPE; and a minimum follow‐up period of 4 weeks post discharge. Dogs were excluded if they had concurrent neurological or orthopedic conditions, or any medical condition affecting rest requirements, any previous spinal surgery, incomplete medical records, or incomplete follow‐up data.

Data collected for each case included breed, age, sex, weight, presenting complaint, whether a triggering event was witnessed, neurological examination findings, neuroanatomical localization, lateralization, spinal hyperesthesia, MRI findings, medications used while hospitalized, additional diagnostic testing performed, days hospitalized, discharge instructions, any incontinence at time of discharge, and re‐evaluation findings.

After MRI, the location of the lesion in terms of the affected intervertebral disc space was recorded, and each case was further characterized according to reported diagnostic criteria as ANNPE, FCE, or HNPE. Any dogs that were euthanized before discharge were excluded. Neurological grade at presentation and re‐evaluation was recorded in accordance with a previously reported six‐point neurological scoring system in dogs, the modified Frankel scoring system (Table 1) [16]. Improvement at re‐evaluation was defined as an improvement of at least one neurological grade.

**TABLE 1:** Classification of neurological grading system (modified Frankel scoring system) neurological grade.

Neurological grade	Description
0	Normal. No neurological abnormalities
1	Pain only
2	Ambulatory paresis
3	Non‐ambulatory paresis
4	Plegic. Deep pain sensation present
5	Plegic. Deep pain sensation absent

For dogs that did not undergo re‐evaluation at the QVSH, in the first instance the referring veterinarian (RV) was contacted to obtain subsequent history. An absence of return to the RV within the first 4 weeks was considered to be consistent with no relapse in clinical signs, as long as subsequent history was available at the RV. Where the RV records were not available, the owner was contacted by phone and asked three questions: Did the dog improve clinically after discharge from QVSH? Did the dog suffer any relapse in clinical signs in the 4 weeks after discharge from QVSH? Did the dog fully recover from the event? Cases were excluded if the follow‐up data from the RV or owner were incomplete.

The remaining included cases then were grouped into “rest” or “exercise” groups based on their discharge letter instructions. Cases where the following wording was used were placed in the “rest” group: rest, strict rest, cage/crate/pen/room rest, restricted exercise/exercise restriction, limit exercise. All other cases were placed into the “exercise” group. Statistical analysis was performed to ensure neurological grade at presentation did not affect which group the animal was assigned to, using a Mann–Whitney test.

## 3 Statistics

Statistical analysis was performed on the two groups to ensure that neurological grade at presentation did not influence the group to which the animals were assigned. Neurological grade at presentation in the two groups (rest vs. exercise) was not normally distributed, based on Shapiro–Wilk and Kolmogorov–Smirnov testing. A Mann–Whitney test was used to compare neurological grades in the two groups, and no significant difference between the two groups was found (*p* = 0.5).

## 4 Results

One‐hundred and twenty cases were identified, of which 62 were excluded because they were diagnosed with another type of neurological disease process or surgical intervention was performed (e.g., Hansen type 1 or 2 intervertebral disc disease, meningomyelitis of unknown origin, vertebral neoplasia). Another 13 were excluded because of incomplete patient records, and 2 were excluded because no imaging was performed to confirm the suspected diagnosis. Forty‐three cases subsequently were included with imaging diagnoses of ANNPE (22), HNPE (7), FCE (11), or FCE/ANNPE (3).

### 4.1 Signalment

Forty‐three dogs fulfilled our inclusion criteria. Twenty‐nine were male (10 intact, 19 neutered) and 14 were female (5 intact, 9 neutered). Twenty‐two different breeds were identified. The most common breeds were Staffordshire bull terrier (5), crossbreed (4), Labrador (4), Cocker spaniel (3), Border collie (3), Chihuahua (3), 2 each of Lhasa apso, Border terrier, Shih tzu, and German shepherd. Other breeds included Lurcher, Bodeguero Andaluz, Greyhound, Boxer, Japanese spitz, Whippet, French bulldog, Shiba inu, Jack Russell terrier, Golden retriever, and Hungarian visla. The age range was 1 year, 1 month to 12 years, 7 months, with a mean of 6 years, 5 months. The average weight was 18.2 kg.

### 4.2 Clinical Presentation

All dogs presented with peracute or acute neurological signs. Seventeen (38%) had experienced a triggering event or trauma such as jumping or falling (12 ANNPE, 1 FCE, 3 HNPE, 1 ANNPE/FCE) and 13 (29%) were reported to have vocalized at the time of onset (10 ANNPE, 3 HNPE). Spinal pain or hyperesthesia was reported in 15/43 cases (6 ANNPE, 3 FCE, 4 HNPE, 2 ANNPE/FCE).

The severity of neurological grade at presentation varied from Grade 2 to 5 (Grade 2 [*n* = 8], Grade 3 [*n* = 24], Grade 4 [*n* = 9], and Grade 5 [*n* = 2]). Twenty‐seven (61%) dogs presented with lateralized neurological signs (16 right, and 11 left), and these lateralized cases consisted of 15 ANNPE, 7 FCE, 2 HNPE, and 3 FCE/ANNPE (Table S1).

### 4.3 Diagnosis and Hospitalization

The most common location was T13‐L1 with 8/43 (18%) of cases (6 ANNPE, 1 HNPE, 1 ANNPE/FCE) occurring at this level (see Table S1). The number of days the patients were hospitalized ranged from 1 to 17 with a mean of 4.2 days.

Three of 43 cases did not survive to discharge. All were diagnosed as thoracolumbar ANNPE cases with neurological grades on presentation of 3, 4, or 5. All were euthanized because of evidence of progressive myelomalacia after hospitalization.

### 4.4 Discharge and Assignment to Groups

Forty dogs survived to discharge. Urinary incontinence was noted at the time of discharge in 6/40 (15%) cases (3 ANNPE, 2 FCE, and 1 HNPE) with 2/40 (5%) showing fecal and urinary incontinence (1 FCE and 1 ANNPE case). One of the six dogs with urinary incontinence showed persistent urinary incontinence at the time of re‐evaluation, 2/6 fully recovered, and urinary function was unknown (presumed recovered) in 3/6. Neither of the dogs with fecal incontinence recovered their voluntary fecal function and subsequently one was euthanized 5 months after discharge because of persistent fecal incontinence despite having improved two neurological grades (from 4 to 2).

Of the 40 dogs that survived to discharge, 17/40 were assigned to the rest group and 23/40 to the exercise group, based on their discharge letter instructions. The mean neurological grade at presentation was 2.9 for the rest group and 3.0 for the exercise group. None of the dogs showed a worsening of neurological grade in the 4‐week period after discharge and no recurrence of signs was seen in either the rest or exercise groups. Two‐thirds of the dogs improved by at least two neurological grades at the time of re‐evaluation and 19/40 (47%) dogs recovered fully after discharge with a neurological grade of 0 at their subsequent re‐evaluation (Table S1).

## 5 Discussion

We found no difference in short‐term (4‐week) recovery or relapse of clinical signs between dogs presenting with ANNPE, HNPE, or FCE and prescribed strict rest and those allowed immediately to exercise, suggesting there is minimal increased risk of further NP extruding after an initial injury when the dog is allowed or encouraged to exercise after such a diagnosis, or that if exercise occurs it does not affect outcome.

After an intervertebral disc extrusion, there is concern that further NP may extrude through the disrupted AF leading to further deterioration in clinical signs, and that this risk may be increased by excessive exercise or movement of the spine [17]. This concern is the reason for the recommendation for a defined period of strict (usually cage confined) rest after a diagnosis of intervertebral disc extrusion in dogs, especially when managed medically. A 4‐week rest period generally is considered appropriate because previous studies have suggested that same site recurrence of intervertebral disc herniation tends to occur within the first 4 weeks [17, 18, 19, 20]. This reason also was why a 4‐week follow‐up period was chosen in our study to minimize the chance of any apparent recurrence of signs being caused by a different neurological episode, such as a new disc extrusion. We hypothesized that because the extruded NP in cases of ANNPE and HNPE is largely non‐degenerate and liquid, the risk to the spinal cord of extrusion of further NP after the initial extrusion should be low. The intra‐discal pressure once ruptured is presumably much lower than in an intact disc, meaning the risk of contusive injury to the spinal cord caused by a sudden escape of NP is likely to be markedly decreased. Our results support this reasoning, although a larger prospective study including more cases in each category would be needed to confirm this suspicion.

Similarly to previous studies of ANNPE and FCE, the most common breeds seen were middle‐aged, non‐chondrodystrophic dogs (e.g., Staffordshire bull terriers, crossbreeds, and Labradors) [11, 12, 21, 22].

Forty‐six percent of cases presented with a history of vocalization or a witnessed triggering event or both. However, because the onset of signs was not witnessed in all cases, this figure may be higher than reported. A noted triggering event such as running or jumping is a well‐known preceding event in FCE, ANNPE, and HNPE cases, although prior case reports suggest a slightly higher frequency than we reported in our study [1, 4, 5, 10].

Asymmetrical or lateralized clinical signs were reported in 58% of cases, which is similar to previous literature on traumatic disc extrusion, ANNPE, and ischemic myelopathy [5, 11, 12]. Spinal hyperesthesia also was present in 15/43 cases (6 ANNPE, 3 FCE, 4 HNPE, 2 FCE/ANNPE), also similar to previous studies [8].

## 6 Limitations

The main limitations of our study were its retrospective nature, small case numbers, and lack of histopathological diagnosis. In terms of our grouping into exercise and rest groups, the exercise group had no standardized exercise regimen to follow. We also have assumed that the owners correctly followed the discharge instructions with respect to resting or exercising their dogs. Also, we assumed that the written discharge instructions were consistent with the verbal instruction provided by the neurologist at the time of discharge.

The duration of hospitalization also may have skewed our results slightly, because those with longer hospitalization times were more likely to have been more strictly cage‐rested during their hospitalization time.

The distribution of diagnoses between the rest and exercise groups was not equal, with a higher proportion of HNPE cases in the rest group and a higher proportion of FCE cases in the exercise group (the distribution of ANNPE cases was very similar between the groups). It might be expected that dogs with FCE would not be at risk of deterioration from exercising, whereas those with HNPE may be more at risk. Our conclusions, therefore, must be interpreted with caution, particularly with respect to HNPE, until a larger study has been performed to corroborate our findings.

Lastly, as seen in Table S1, in some cases assessment of neurological outcome was based on a telephone call with the owner rather than a follow‐up appointment with a veterinarian.

## 7 Conclusion

Given our results, we were able to accept both of our hypotheses. Allowing exercise after confirming ANNPE, FCE, or HNPE as a diagnosis does not increase the risk of a deterioration in clinical signs in the short‐term follow‐up period (4 weeks) after discharge, at least in this group of dogs, with no difference in outcome between rested vs. exercised groups. This conclusion should be interpreted with caution with respect to dogs with HNPE because only a small number of such cases were included in our exercise group.

## Disclosure

Authors declare no off‐label use of antimicrobials.

## Ethics Statement

Approved by the Department of Veterinary Medicine, University of Cambridge, Ethics and Welfare Research, CR753, March 10, 2023.

## Conflicts of Interest

The authors declare no conflicts of interest.

## Supporting information


**Table S1.** Supporting Information.

## References

[1] G. Gandini , S. Cizinauskas , J. Lang , R. Fatzer , and A. Jaggy , “Fibrocartilaginous Embolism in 75 Dogs: Clinical Findings and Factors Influencing the Recovery Rate,” Journal of Small Animal Practice 44, no. 2 (2003): 76–80. 12622472 10.1111/j.1748-5827.2003.tb00124.x

[2] L. De Risio , “A Review of Fibrocartilaginous Embolic Myelopathy and Different Types of Peracute Non‐Compressive Intervertebral Disk Extrusions in Dogs and Cats,” Frontiers in Veterinary Science 2 (2015): 24. 26664953 10.3389/fvets.2015.00024 PMC4672181

[3] J. Fenn , R. Drees , H. A. Volk , and S. De cker , “Inter‐ and Intraobserver Agreement for Diagnosing Presumptive Ischemic Myelopathy and Acute Noncompressive Nucleus Pulposus Extrusion in Dogs Using Magnetic Resonance Imaging,” Veterinary Radiology & Ultrasound 57, no. 1 (2016): 33–40. 26306004 10.1111/vru.12289

[4] S. Decker and J. Fenn , “Acute Herniation of Nondegenerate Nucleus Pulposus: Acute Noncompressive Nucleus Pulposus Extrusion and Compressive Hydrated Nucleus Pulposus Extrusion,” Veterinary Clinics of North America. Small Animal Practice 48 (2018): 95–109. 28964544 10.1016/j.cvsm.2017.08.004

[5] E. Beltran , “Acute Hydrated Non‐Compressive Nucleus Pulposus Extrusion: What Do We Know So Far?,” Veterinary Record 181 (2017): 591–593. 29192043 10.1136/vr.j5494

[6] C. Ros , C. De La Fuente , S. Rodenas , R. Novellas , J. Viu , and S. Añor , “Myelographic and Low‐Field Magnetic Resonance Imaging Findings in Dogs With Presumptive Acute Hydrated Non‐Compressive Nucleus Pulposus Extrusion,” Veterinary Record 181, no. 22 (2017): 594–599. 29051312 10.1136/vr.104201

[7] L. Mari , S. Behr , A. Shea , et al., “Outcome Comparison in Dogs With a Presumptive Diagnosis of Thoracolumbar Fibrocartilaginous Embolic Myelopathy and Acute Non‐Compressive Nucleus Pulposus Extrusion,” Veterinary Record 181, no. 11 (2017): 293. 28784693 10.1136/vr.104090

[8] J. Fenn , R. Drees , H. A. Volk , and S. de Decker , “Comparison of Clinical Signs and Outcomes Between Dogs With Presumptive Ischemic Myelopathy and Dogs With Acute Non‐Compressive Nucleus Pulposus Extrusion,” Journal of the American Veterinary Medical Association 249, no. 7 (2016): 767–775. 27654163 10.2460/javma.249.7.767

[9] J. Nessler , C. Flieshardt , J. Tünsmeyer , R. Dening , and A. Tipold , “Comparison of Surgical and Conservative Treatment of Hydrated Nucleus Pulposus Extrusion in Dogs,” Journal of Veterinary Internal Medicine 32, no. 6 (2018): 1989–1995. 30267615 10.1111/jvim.15304 PMC6271322

[10] K. V. Kristiansen , H. Schmökel , and S. Vermeire , “Hydrated Nucleus Pulposus Extrusion in Dogs: Thoracolumbar Compared to Cervical Cases,” Veterinary and Comparative Orthopaedics and Traumatology 35, no. 3 (2022): 152–156. 35008122 10.1055/s-0041-1740608

[11] W. M. McKee , C. J. Downes , J. J. Pink , and T. J. Gemmill , “Presumptive Exercise‐Associated Peracute Thoracolumbar Disc Extrusion in 48 Dogs,” Veterinary Record 166, no. 17 (2010): 523–528. 20418513 10.1136/vr.b4823

[12] L. Risio , V. Adams , R. Dennis , and F. J. McConnell , “Association of Clinical and Magnetic Resonance Imaging Findings With Outcome in Dogs With Presumptive Acute Non‐Compressive Nucleus Pulposus Extrusion: 42 Cases,” Journal of the American Veterinary Medical Association 234, no. 4 (2009): 495–504. 19222359 10.2460/javma.234.4.495

[13] D. Gouveia , A. Cardoso , C. Carvalho , et al., “Influence of Spinal Shock on the Neurorehabilitation of ANNPE Dogs,” Animals 12, no. 12 (2022): 1557. 35739893 10.3390/ani12121557 PMC9219513

[14] M. Dorn , “Crate Confinement of Dogs Following Orthopaedic Surgery. Part 1: Benefits and Possible Drawbacks,” Companion Animals 22, no. 7 (2017): 368–376.

[15] F. Watson , “Practical Considerations When Treating Pets of Owners With no Fixed Abode: Treatment Priorities, Implementation and Compliance,” (Birmingham, 2020), BSAVA Congress Proceedings.

[16] J. Hermansen , M. Kuricová , and T. Lipták , “Intervertebral Disc Disease in Dogs—The Relationship Between Recovery and Timing of Surgery,” Folia Veterinaria 66, no. 3 (2022): 54–59.

[17] S. Dhupa , N. Glickman , and D. J. Waters , “Reoperative Neurosurgery in Dogs With Thoracolumbar Disc Disease,” Veterinary Surgery 28, no. 6 (1999): 421–428. 10582738 10.1111/j.1532-950x.1999.00421.x

[18] S. Longo , S. A. Gomes , C. Briola , et al., “Association of Magnetic Resonance Assessed Disc Degeneration and Late Clinical Recurrence in Dogs Treated Surgically for Thoracolumbar Intervertebral Disc Extrusions,” Journal of Veterinary Internal Medicine 35, no. 1 (2021): 378–387. 33283382 10.1111/jvim.15989 PMC7848362

[19] E. Suiter , N. Grapes , L. Martin‐Garcia , S. De cker , R. Gutierrez‐Quintana , and A. Wessmann , “MRI and Clinical Findings in 133 Dogs With Recurrent Deficits Following Intervertebral Disc Extrusion Surgery,” Veterinary Record 193, no. 5 (2023): e2992. 37247382 10.1002/vetr.2992

[20] N. Olby , S. Moore , B. Brisson , et al., “ACVIM Consensus Statement on Diagnosis and Management of Acute Canine Thoracolumbar Intervertebral Disc Extrusion,” Journal of Veterinary Internal Medicine 36 (2022): 1570–1596. 35880267 10.1111/jvim.16480 PMC9511077

[21] K. A. Bartholomew , K. E. Stover , N. J. Olby , and S. A. Moore , “Clinical Characteristics of Canine Fibrocartilaginous Embolic Myelopathy (FCE): A Systematic Review of 393 Cases (1973–2013),” Veterinary Record 179 (2016): 650. 27682506 10.1136/vr.103863

[22] Y. Chang , R. Dennis , S. R. Platt , and J. Penderis , “Magnetic Resonance Imaging of Traumatic Intervertebral Disc Extrusion in Dogs,” Veterinary Record 160, no. 23 (2007): 795–799. 17558027 10.1136/vr.160.23.795

