# Supplementary material for: Exercise Restriction Does Not Change Outcome in Dogs After Diagnosis of Acute Non‐Compressive Nucleus Pulposus Extrusion, Fibrocartilaginous Embolism, or Hydrated Nucleus Pulposus Extrusion
Source: J Vet Intern Med. 2025 Jun 3;39(4):e70135. doi: 10.1111/jvim.70135 (PMC12131279; doi:10.1111/jvim.70135)
Supplement: Supplementary file 1 — Table S1. Supporting Information. [file JVIM-39-e70135-s001.docx]

Supplementary Table: Summary of Included cases data set

| **Dog** | **Breed** | **Age** | **Sex** | **Onset** | **Neurological grade at presentation** | **Lateralised** | **Spinal pain** | **Neuro-localisation** | **Diagnosis** | **No of days hospitalised** | **Rest vs Exercise** | **Neurological grade at final re-check** | **Number of neurological grades improved** | **Outcome confirmed at recheck?** | **Persistent Incontinence?** |
| --- | --- | --- | --- | --- | --- | --- | --- | --- | --- | --- | --- | --- | --- | --- | --- |
| 1 | Labrador | 4yr | MN | Acute | 4 | Yes | No | T3-L3 | T12-13 ANNPE | 7 | Exercise | 2 | 2 | Y | No |
| 2 | Labrador | 12yr7mo | ME | Acute | 3 | No | Yes | C1-C5 | C4-5 HNPE | 4 | Rest | 3 | 0 | Y | No |
| 3 | Hungarian Visla | 7yr8mo | FE | Acute | 4 | No | No | C6-T2 | C4-5 HNPE | 4 | Rest | 2 | 2 | Y | No |
| 4 | Chihuahua | 3yr5mo | MN | Peracute | 3 | No | No | C6-T2 | C5-7 FCE | 6 | Exercise | 2 | 1 | Contacted Owner | No |
| 5 | Italian Greyhound | 4yr6mo | FE | Peracute | 3 | Yes | Yes | C1-C5 | C4-5 ANNPE | 3 | Rest | 2 | 1 | Y | No |
| 6 | Labrador | 8yr2mo | FN | Peracute | 4 | No | Yes | T3-L3 | T12-T13 HNPE | 4 | Rest | 2 | 2 | Y | No |
| 7 | Staffordshire bull terrier | 4yr4mo | ME | Peracute | 3 | Yes | No | T3-L3 | T12-T13 ANNPE | 2 | Rest | 0 | 3 | Y | No |
| 8 | Border terrier | 8yr | FE | Acute | 3 | Yes | Yes | T3-L3 | T13-L1 HNPE | 4 | Rest | 2 | 1 | Y | No |
| 9 | Chihuahua | 1yr8mo | FN | Peracute | 3 | Yes | Yes | C6-T2 | C5-7 FCE | 4 | Exercise | 2 | 1 | Y | No |
| 10 | Lhasa Apso | 8yr10mo | FN | Peracute | 3 | Yes | Yes | T3-L3 | T10-L3 FCE | 2 | Exercise | 2 | 1 | Y | No |
| 11 | Shih tzu | 4yr10mo | FN | Acute | 3 | Yes | No | T3-L3 | T5 & L1-2 FCE | 2 | Rest | 0 | 3 | Contacted Owner | No |
| 12 | Chihuahua | 3yr6mo | MN | Acute | 3 | Yes | No | C6-T2 | T1-3 FCE | 2 | Exercise | 0 | 3 | Y | No |
| 13 | Staffordshire bull terrier | 8yr | FN | Acute | 2 | Yes | No | T3-L3 | T11-L1 FCE | 2 | Exercise | 2 | 0 | Y | No |
| 14 | Shih Tzu | 5yr11mo | MN | Acute | 3 | Yes | No | C6-T2 | C6-7 FCE | 5 | Exercise | 2 | 1 | Y | No |
| 15 | Miniature schnauzer | 7yr | MN | Acute | 5 | No | No | T3-L3 | T12-L1 FCE | 17 | Exercise | 2 | 3 | Y | Faecal |
| 16 | Lurcher | 2yr3mo | MN | Peracute | 3 | No | Yes | C1-C5 | C2-3 FCE | 2 | Exercise | 0 | 3 | Y | No |
| 17 | Border Terrier | 9yr | ME | Acute | 2 | No | Yes | C1-C5 | C3-4 HNPE | 2 | Rest | 0 | 2 | Contacted Owner | No |
| 18 | French bulldog | 3yr | ME | Acute | 3 | No | Yes | L4-S3 | L3-4 ANNPE | 1 | PTS | 3 | 0 | n/a | N/a |
| 19 | Japanese spitz | 5yr1mo | MN | Peracute | 3 | Yes | No | C1-T2 | C2-3 FCE | 4 | Exercise | 2 | 1 | Y | No |
| 20 | Bodeguero Andaluz | 11yr | MN | Peracute | 3 | Yes | Yes | C6-T2 | C2-3 FCE/ANNPE | 4 | Exercise | 2 | 1 | Y | No |
| 21 | German Shephard | 6yr5mo | ME | Acute | 4 | No | No | L4-S3 | L3-4 FCE | 3 | Rest | 2 | 2 | Y | Urinary |
| 22 | Boxer | 6yr5mo | FE | Peracute | 3 | Yes | No | T3-L3 | T13-L1 ANNPE | 4 | Exercise | 0 | 3 | Y | No |
| 23 | Lurcher | 4yr4mo | FN | Peracute | 3 | No | No | C1-C5 | C3-4 ANNPE | 2 | Exercise | 0 | 3 | Y | No |
| 24 | Border collie | 7yr10mo | MN | Peracute | 2 | Yes | No | T3-L3 | T13-L1 ANNPE | 1 | Exercise | 0 | 2 | Y | No |
| 25 | Border collie | 6yr | FE | Peracute | 4 | Yes | Yes | T3-L3 | T13-L1 ANNPE | 6 | Exercise | 2 | 2 | Y | Faecal |
| 26 | Cocker spaniel | 6yr7mo | ME | Acute | 2 | Yes | Yes | T3-L3 | T13-L1 ANNPE | 2 | Rest | 0 | 2 | Y | No |
| 27 | Staffordshire bull terrier | 4yr6mo | MN | Acute | 2 | Yes | No | T3-L3 | T12-13 ANNPE | 1 | Exercise | 0 | 2 | Y | No |
| 28 | Cocker spaniel | 1yr1mo | MN | Peracute | 3 | Yes | No | C6-T2 | C7-T1 ANNPE | 2 | Rest | 2 | 1 | Contacted Owner | No |
| 29 | Labradoodle | 8yr2mo | ME | Peracute | 3 | Yes | No | C1-T2 | C5-6 ANNPE | 3 | Exercise | 0 | 3 | Y | No |
| 30 | Miniature schnauzer | 10yr9mo | MN | Acute | 3 | Yes | No | T3-L3 | T13-L1 ANNPE | 1 | Rest | 0 | 3 | Y | No |
| 31 | Labrador | 7yr11mo | FN | Peracute | 4 | Yes | No | T3-L3 | T12-13 ANNPE | 8 | Exercise | 0 | 4 | Y | No |
| 32 | Jack Russell terrier | 10yr9mo | MN | Acute | 3 | No | No | C1-C5 | C3-4 HNPE | 4 | Rest | 0 | 3 | Contacted Owner | No |
| 33 | Border collie | 8yr7mo | MN | Peracute | 4 | No | No | T3-L3 | T12-13 ANNPE | 5 | PTS | 5 | -1 | n/a | n/a |
| 34 | Cocker spaniel | 7yr8mo | FN | Acute | 4 | No | No | T3-L3 | T12-13 ANNPE | 9 | Rest | 2 | 2 | Y | No |
| 35 | Shiba Inu | 9yr | MN | Acute | 2 | Yes | Yes | T3-l3 | T13-L1 ANNPE | 8 | Rest | 0 | 2 | Contacted Owner | No |
| 36 | Staffordshire bull terrier | 7yr | MN | Acute | 2 | Yes | No | T3-L3 | L2-3 FCE/ANNPE | 2 | Rest | 0 | 2 | Y | No |
| 37 | Lhasa Apso | 5yr1mo | MN | Peracute | 5 | Yes | No | T3-l3 | L2-3 ANNPE | 1 | PTS | 5 | 0 | n/a | n/a |
| 38 | Golden retriever | 9yr1mo | MN | Peracute | 3 | Yes | No | T3-L3 | L1-2 HNPE | 3 | Exercise | 2 | 1 | Y | No |
| 39 | Chihuahua | 6yr6mo | ME | Peracute | 3 | No | No | C1-T2 | C4-5 ANNPE | 5 | Exercise | 0 | 3 | Y | No |
| 40 | German Shepard | 2yr3mo | ME | Acute | 2 | No | No | T3-L3 | T7-8 ANNPE | 2 | Rest | 2 | 0 | Contacted Owner | No |
| 41 | Staffordshire bull terrier | 4yr1mo | ME | Peracute | 4 | No | Yes | T3-L3 | T13-L1 FCE/ANNPE | 12 | Exercise | 0 | 4 | Y | No |
| 42 | Crossbreed | 8yr | FN | Peracute | 3 | No | Yes | L4-S3 | L4-L5 ANNPE | 7 | Exercise | 0 | 3 | Contacted Owner | No |
| 43 | Whippet | 7yr | MN | Acute | 3 | Yes | No | C1-C5 | C4-5 ANNPE | 8 | Exercise | 2 | 1 | Y | No |
